# Superior Approach to Sacroiliac (SI) Joint Injection: A Case Report

**DOI:** 10.7759/cureus.73997

**Published:** 2024-11-19

**Authors:** Sai Charan Chilumula, Irene Mavrakakis

**Affiliations:** 1 Emergency Medicine, Bayhealth Hospital, Dover, USA; 2 Interventional Pain, Bayhealth Hospital, Dover, USA

**Keywords:** interventional pain medicine, lumbo-sacral pain, sacroilitis, si joint injection, treating low back pain

## Abstract

The sacroiliac joint is a well-recognized source of chronic lower back pain. In interventional management, the inferior approach to intra-articular sacroiliac joint injection has been more extensively studied and commonly utilized in clinical practice. This case report presents the superior one-third approach as an alternative technique for treating sacroiliac joint pain in a patient, highlighting its potential efficacy and clinical relevance.

## Introduction

The sacroiliac (SI) joint has been implicated as the primary source of pain generator in approximately 10%-30% of patients with chronic low back pain [1-4]. Effective treatment can include conservative management such as non-steroidal anti-inflammatory drugs (NSAIDs), physical therapy, lifestyle modifications, interventional treatment using intraarticular injections of local anesthetics and corticosteroids, and surgical options such as joint arthrodesis in refractory cases.

For the intra-articular approach to injections, conventionally the inferior one-third approach is more commonly accepted. This approach may have limitations due to anatomic variances, and complexity due to joint degeneration, narrowing or osteophytes [5]. In such circumstances, alternative approaches can be implemented. A case with a superior approach has been described in this report.

## Case presentation

**Figure 1:**
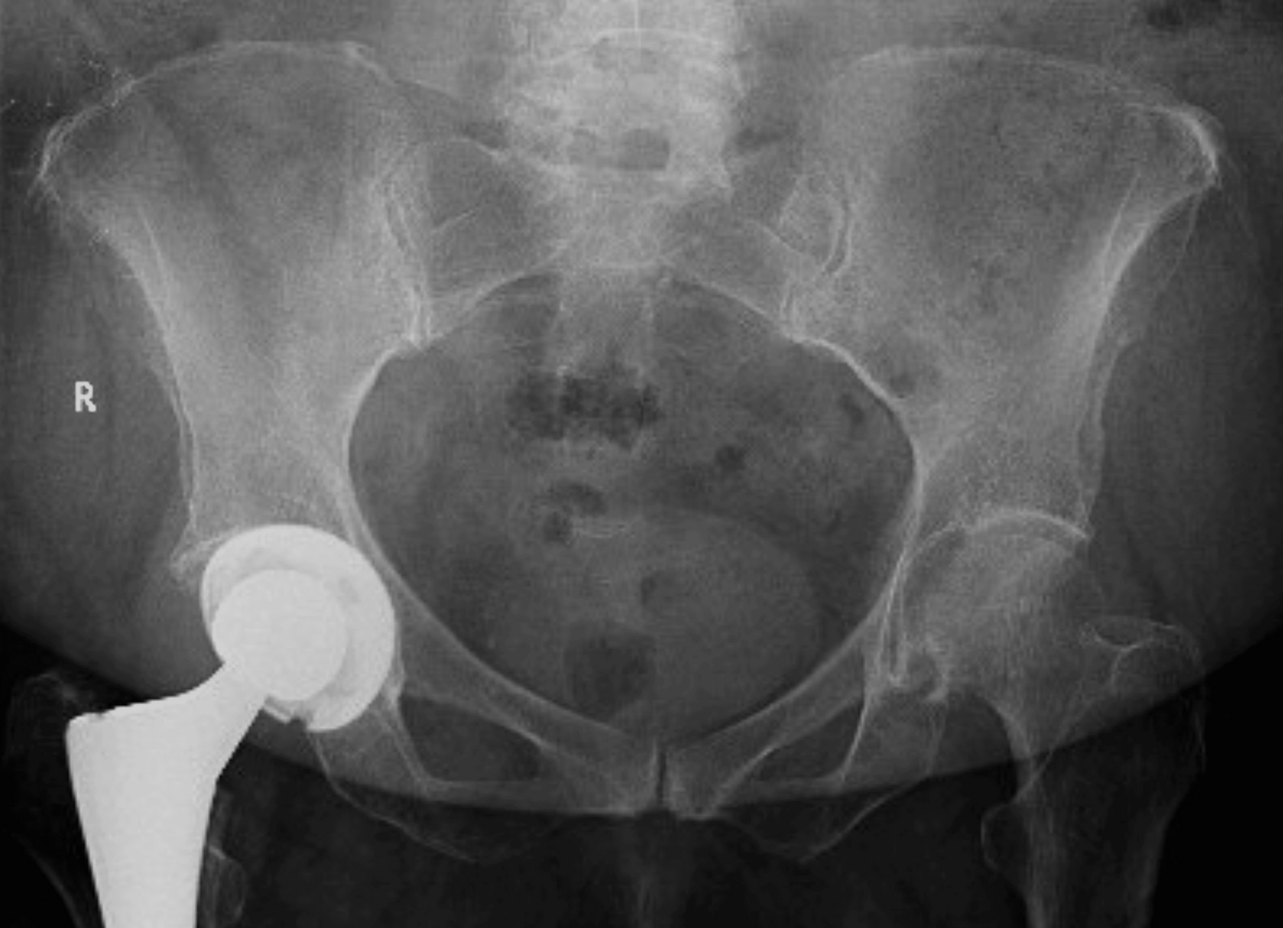
Degenerative changes noted in bilateral sacroiliac joint

A 72-year-old woman with a history of chronic lower and sacroiliac pain presented for care. Patient workup was positive for the Fortin finger test and sacroiliac arthropathy in imaging (Figure 1). Considering the patient's previous therapeutic response, the patient was scheduled for a bilateral sacroiliac injection.

Before the injection, the patient-reported pain level was an 8 out of 10 on a numeric scale.

During the therapeutic procedure, an aseptic technique was used with the patient in the prone position and a pillow underneath the abdomen on a fluoroscopy table. The C-arm anterior-posterior view identified the anterior and posterior sacroiliac joint lines. The skin and subcutaneous tissue were infiltrated with 2 ml of 1% lidocaine with a 25-gauge needle. A 10 cm, 22-gauge spinal needle was inserted and directed towards the upper one-third of the joint at an angle of about 30%-45%. The space formed between the ileum and the lateral border of the sacral ala was targeted correcting with fluoroscopy as needed. The position was confirmed with a 40%-50% contralateral oblique view after negative aspiration. Then, 1 ml of 1% lidocaine and 40 mg of methylprednisolone in saline (total volume of 2 ml) was injected. Then using the same needle, periarticular injection is performed. The needle is withdrawn cranially with positioning confirmed with a contralateral oblique view. Then, 1 ml of 1% lidocaine and 20 mg of methylprednisolone were injected in saline (total volume of 1.5 ml). This was repeated for bilateral SI joints. The procedure only required one successful attempt. The patient tolerated the procedure well with no complications. The patient denied lower extremity numbness, paresthesias, or weakness. The patient was able to ambulate and was discharged to home.

The patient followed up in two weeks with satisfactory therapeutic pain relief. The patient rated pain level improved from an 8 out of 10 to a 3 out of 10 on a numeric pain scale and a self-reported 90% subjective pain relief.

## Discussion

The inferior approach to sacroiliac (SI) joint injection is often preferred by clinicians due to a combination of technical and anatomical factors that make it safer and more straightforward than the superior approach. The SI joint itself has two distinct limbs: the inferior and superior limbs. The inferior limb is both longer and more accessible, which provides a more defined and reproducible entry path for needle insertion. This anatomical configuration simplifies the process of reaching the joint space, allowing for more consistent results across patients [6].

In contrast, the superior limb of the SI joint is situated closer to the sacral foramina, where the sacral nerves exit. This proximity increases the risk of inadvertent nerve injury, which can lead to significant complications such as nerve damage, pain exacerbation, or sensory disturbances. As a result, clinicians typically avoid the superior limb approach unless there are specific indications that justify the potential risk.

While studies evaluating the superior approach to SI joint injection exist, they predominantly focus on short-term outcomes, often assessing immediate or early post-procedure pain relief and functionality. This limited focus underscores a gap in the literature regarding the long-term efficacy and safety of the superior approach. Future research should aim at addressing this by conducting long-term studies on the superior approach, evaluating not only the sustained pain relief but also the potential risks of cumulative nerve irritation or damage over time. Expanding the research in this area could offer valuable insights into whether the superior approach could be a viable alternative for certain patient populations and conditions, provided its safety and efficacy can be demonstrated over a prolonged period.

In cases where anatomical changes like osteophytes or joint narrowing impede the needle’s access to the SI joint through the inferior approach, alternative methods become essential. Osteophyte formation or narrowing of the joint space, often due to degenerative changes, can obstruct the entry pathway that would typically allow for straightforward needle placement in the inferior limb. This situation presents a unique challenge for clinicians, as the restricted space increases the risk of incomplete injections or procedural complications. 

The superior one-third injection technique has shown promise as an effective alternative in such cases. According to research by Park et al., this technique has achieved a notable 90% success rate in patients where the inferior approach could not be successfully implemented. This success rate suggests that the superior approach offers a viable solution in situations where conventional methods fall short, providing a dependable route to administer intra-articular injections in anatomically challenging cases [7].

Studies have further demonstrated that the superior approach is non-inferior to the inferior approach in terms of key procedural outcomes, including contrast distribution, procedural success rates, and complication rates [8]. These findings highlight the comparable safety and effectiveness of the superior approach, indicating that it can deliver results on par with those of the more commonly used inferior route. Additionally, the superior approach has been consistently associated with significant reductions in pain and disability scores, underscoring its therapeutic benefits for patients suffering from SI joint dysfunction [9,10].

Considering these findings, the superior approach may serve as a valuable alternative or even a primary option in treating SI joint dysfunction, particularly in patients with complex joint anatomy. By broadening the scope of injection techniques, clinicians can tailor their approach based on individual anatomical considerations, thereby enhancing procedural success and patient outcomes.

## Conclusions

Sacroiliac joint injections have been a mainstay in the treatment of SI joint pain for several decades, with the inferior approach being the more commonly accepted technique. This preference may be attributed to the fact that SI joint pain often originates from the inferior aspect of the joint, as it is more frequently symptomatic based on referred pain patterns. However, it is important to acknowledge the limitations of the inferior approach, particularly in cases involving degenerated joints with narrowing, osteophytes, or prior unsuccessful attempts. In such instances, the superior approach should be considered due to its high success rate, comparable efficacy, and consistent outcomes in managing sacroiliac joint pain.
